# Cause of death in patients diagnosed with esophageal cancer in Sweden: a population-based study

**DOI:** 10.18632/oncotarget.15270

**Published:** 2017-02-11

**Authors:** Shao-Hua Xie, Karl Wahlin, Jesper Lagergren

**Affiliations:** ^1^ Upper Gastrointestinal Surgery, Department of Molecular Medicine and Surgery, Karolinska Institutet, Karolinska University Hospital, Sweden; ^2^ Division of Cancer Studies, King’s College London, United Kingdom

**Keywords:** esophageal cancer, prognosis, mortality, cause of death, Sweden

## Abstract

**Background:**

Esophageal cancer carries a poor prognosis with an overall 5-year survival of less than 20%. However, the causes of death in patients with esophageal cancer have not been well described.

**Methods:**

This nationwide, population-based cohort study included 18 229 esophageal cancer patients who were diagnosed between 1961 and 2014 in Sweden. We assessed the distribution of main causes of death in patients with esophageal cancer and used competing-risks regression to compare the cause-specific risks of death across sexes, ages at diagnosis, and calendar periods of diagnosis.

**Results:**

A total of 16 938 (92.9%) patients died during follow-up. Esophageal cancer accounted for 79.5% of all reported deaths. Other major causes of death were non-esophageal cancers (9.8%), ischemic heart disease or cerebrovascular disease (4.2%) and respiratory diseases (1.3%). Female patients had a lower risk of death from esophageal cancer (sub-hazard ratio [SHR]=0.90, 95% confidence interval [CI]: 0.87, 0.94), which was more pronounced in patients with squamous cell carcinoma (SHR=0.85, 95% CI: 0.81, 0.89). Risks of death from esophageal cancer and other cancers in patients who were diagnosed in more recent calendar periods were lower than in those diagnosed in earlier periods.

**Conclusions:**

Patients diagnosed with esophageal cancer are more likely to die from this cancer than from other causes. However, these patients also face considerable risk of death from other cancers, ischemic heart disease, cerebrovascular disease, and respiratory diseases. These common causes of death should be taken into consideration in esophageal cancer management.

## INTRODUCTION

Esophageal cancer is the eighth most common type of malignancy and the sixth leading cause of cancer death globally. [1, 2] There are two main histological types, i.e., esophageal squamous cell carcinoma (ESCC) and esophageal adenocarcinoma (EAC). Although ESCC accounts for approximately 90% of all cases of esophageal cancer worldwide, the incidence of EAC has been rapidly increasing in many regions, including Europe and North America, during the past four decades. [3, 4]

Esophageal cancer carries a poor prognosis with the overall 5-year survival following diagnosis lower than 20%. [3–5] Understanding the specific causes of death in patients with this cancer is essential for improving the prognosis, but reliable estimates of these causes are largely lacking. Previous studies have indicated a considerable proportion of deaths attributable to non-cancer conditions in patients with esophageal cancer. [6–8] A high incidence of secondary malignancies in patients with esophageal cancer has also been noted. [9, 10] However, these were mainly observations based on relatively small groups of patients, and the causes of death in patients with esophageal cancer have not been well described.

To improve our understanding of the excess mortality in relation to esophageal cancer, we performed this population-based study to assess causes of death in patients diagnosed with esophageal cancer in Sweden over more than 5 decades. A competing-risks model was applied to compare cause-specific risks of death by sex, age at diagnosis, and calendar period of diagnosis.

## RESULTS

### Patients

This study included 18 229 patients diagnosed with esophageal cancer, including 10 230 (56 %) cases of ESCC, 5 140 (28 %) cases of EAC, and the remaining cases were diagnosed with other (*n* = 2 189, 12 %) or unspecified (*n* = 670, 4 %) histology. Males accounted for 81.2% of all EAC cases and 66.1% of all ESCC cases. The mean (standard deviation) age at diagnosis was 70.1 (10.8) years. More detailed distribution of all cases of esophageal cancer by sex, age at diagnosis, calendar period of diagnosis, and follow-up time is presented in Table 1.

**Table 1:** Basic characteristics of all patients with esophageal cancer diagnosed in Sweden in 1961-2014, number (%)

Characteristic	Squamous cell carcinoma	Adenocarcinoma	Total esophageal cancer
Total	10 230 (100.0)	5 140 (100.0)	18 229 (100.0)
Sex			
Male	6 763 (66.1)	4 173 (81.2)	12 867 (70.6)
Female	3 467 (33.9)	967 (18.8)	5 362 (29.4)
Age at diagnosis, years			
<50	319 (3.1)	212 (4.1)	630 (3.5)
50-59	1 378 (13.5)	717 (13.9)	2 390 (13.1)
60-69	3 189 (31.2)	1 491 (29.0)	5 344 (29.3)
70-79	3 541 (34.6)	1 622 (31.6)	6 161 (33.8)
≥80	1 803 (17.6)	1 098 (21.4)	3 704 (20.3)
Mean (standard deviation)	69.6 (10.4)	69.8 (11.2)	70.1 (10.8)
Calendar period of diagnosis			
1961-1970	1 564 (15.3)	231 (4.5%)	2 376 (13.0)
1971-1980	1 885 (18.4)	390 (7.6)	2 818 (15.5)
1981-1990	2 190 (21.4)	484 (9.4)	3 070 (16.8)
1991-2000	2 099 (20.5)	1 004 (19.5)	3 631 (19.9)
2001-2014	2 492 (24.4)	3 031 (59.0)	6 334 (34.7)
Length of follow-up, years			
≤1	7 429 (72.6)	3 419 (66.5)	12 838 (70.4)
1-5	2 180 (21.3)	1 356 (26.4)	4 050 (22.2)
>5	621 (6.1)	365 (7.1)	1 341 (7.4)

### Causes of death

Table 2 shows the distribution of cause-specific deaths in patients diagnosed with esophageal cancer by duration of follow-up. A total of 16 938 (92.9%) patients died during follow-up, among which 12 487 (73.8% of all deaths) occurred within 1 year of diagnosis. The median survival following diagnosis estimated from 16 499 patients with complete follow-up was 179 days (95% confidence interval [CI]: 175, 183), which did not differ by histological type (ESCC, 185 days, 95% CI: 180, 191; EAC, 194 days, 95% CI: 185, 204). The median survival time in female patients (192 days, 95% CI: 184, 201) was slightly longer than in males (175 days, 95% CI: 170, 179).

**Table 2:** Causes of death by duration of follow-up in patients with esophageal cancer in Sweden in 1961-2014

Mortality status	Duration of follow-up		
	≤1 year Number (%)	1-5 years Number (%)	>5 years Number (%)
Total *	18 229	5 391	1 341
Alive throughout follow-up ^† ‡^	351 (1.9)	415 (7.7)	525 (39.1)
All deaths ^‡^	12 487 (68.5)	3 635 (67.4)	816 (60.9)
Cause-specific death ^‡^			
Esophageal cancer	10 415 (57.1)	2 849 (52.8)	210 (15.7)
Cancers other than esophageal cancer	1 091 (6.0)	385 (7.1)	181 (13.5)
Infectious and parasitic diseases	21 (0.1)	11 (0.2)	7 (0.5)
Diseases of the respiratory system	113 (0.6)	49 (0.9)	64 (4.8)
Chronic obstructive pulmonary disease	52 (0.3)	21 (0.4)	29 (2.2)
Influenza and pneumonia	43 (0.2)	22 (0.4)	24 (1.8)
Ischemic heart disease	320 (1.8)	127 (2.4)	128 (9.5)
Cerebrovascular disease	60 (0.3)	41 (0.8)	36 (2.7)
Diabetes	23 (0.1)	1 (<0.1)	2 (0.1)
Dementia and Alzheimer's disease	2 (0.1)	1 (<0.1)	17 (1.3)
External causes, including suicide	14 (0.1)	9 (0.2)	16 (1.2)
Suicide	6 (<0.1)	2 (<0.1)	2 (0.1)
Other specified	428 (2.3)	162 (3.0)	155 (11.6)

*Number of patients alive at the beginning of the follow-up window.

^†^ Patients alive at the beginning of the follow-up window who did not die during the specified period of follow-up.

‡ Percentage of all patients alive at the beginning of the follow-up window.

Esophageal cancer was the most common cause of death; nearly three quarters of all patients diagnosed with esophageal cancer (73.9%, *n* = 13 474) died from this disease. Non-esophageal cancers were the second most common cause of death, accounting for 1 657 (9.8%) of all deaths. Among all reported deaths, 712 (4.2%) were due to ischemic heart disease or cerebrovascular disease, and 226 (1.3%) were due to respiratory diseases. Deaths from causes other than esophageal cancer were more common in patients with longer follow-up (longer survival). For example, 13.5% of all patients who were followed up for over 5 years died from non-esophageal cancers, while 6.0% of patients with ≤ 1 year follow-up died from these cancers (Table 2).

Among all deaths from non-esophageal cancers, 982 (59.3) were due to cancers of digestive organs, 155 (9.4%) due to cancers of the lip, oral cavity, and pharynx, and 145 (8.8%) due to lung cancer (Table 3). Gastric cancer was the single most common cause (*n* = 851, 51.4%) of death from non-esophageal cancers (Table 3).

**Table 3:** Deaths from non-esophageal cancers in patients diagnosed with esophageal cancer in Sweden in 1961-2014

Sites	Total esophageal cancer Number (%)	Squamous cell carcinoma Number (%)	Adenocarcinoma Number (%)
Total	1 657 (100.0)	686 (100.0)	694 (100.0)
Lip, oral cavity, and pharynx	155 (9.4)	134 (19.5)	2 (0.3)
Digestive organs ^†^	982 (59.3)	275 (40.1)	577 (83.1)
Stomach	851 (51.4)	216 (31.5)	535 (77.1)
Colon, rectum, and anus	44 (2.7)	21 (3.1)	14 (2.0)
Liver and biliary passages	25 (1.5)	10 (1.5)	6 (0.9)
Pancreas	42 (2.5)	24 (3.5)	9 (1.3)
Lung (incl. trachea and bronchus)	145 (8.8)	92 (13.4)	22 (3.2)
Breast	25 (1.5)	11 (1.6)	7 (1.0)
Uterus	7 (0.4)	5 (0.7)	1 (0.1)
Ovary	5 (0.3)	3 (0.4)	1 (0.1)
Prostate	52 (3.1)	15 (2.2)	23 (3.3)
Testis	2 (0.1)	1 (0.1)	0 (0.0)
Bladder	8 (0.5)	3 (0.4)	1 (0.1)
Kidney	14 (0.8)	7 (1.0)	4 (0.6)
Thyroid	9 (0.5)	5 (0.7)	0 (0.0)
Hodgkin lymphoma	1 (0.1)	1 (0.1)	0 (0.0)
Non-Hodgkin lymphoma	13 (0.8)	4 (0.6)	5 (0.7)
Leukemia	12 (0.7)	6 (0.9)	4 (0.6)
Other and unspecified	227 (13.7)	124 (18.1)	47 (6.8)

Stratified analyses by histological type showed that compared with patients with ESCC, patients diagnosed with EAC were less likely to die from esophageal cancer but more likely to die from other cancers (Table 4).

**Table 4:** Distribution of causes of death by duration of follow-up in patients with esophageal adenocarcinoma and esophageal squamous cell carcinoma in Sweden, 1961-2014

Mortality status	Adenocarcinoma			Squamous cell carcinoma		
	≤1 year	1-5 years	>5 years	≤1 year	1-5 years	>5 years
Number of individuals at risk *	5 140	1 721	365	10 230	2 801	621
Alive throughout follow-up, n (%) ^†^	185 (3.6)	202 (11.7)	206 (56.4)	118 (1.2)	120 (4.3)	156 (25.1)
All deaths, n (%) ^‡^	3 234 (62.9)	1 154 (67.1)	159 (43.6)	7 311 (71.5)	2 060 (73.5)	465 (74.9)
Cause-specific death, n (%) ^‡^						
Esophageal cancer	2 532 (49.3)	856 (49.7)	44 (12.1)	6 308 (61.7)	1 723 (61.5)	129 (20.8)
Cancers other than esophageal cancer	450 (8.8)	197 (11.4)	47 (12.9)	458 (4.5)	133 (4.7)	95 (15.3)
Infectious and parasitic diseases	8 (0.2)	6 (0.3)	2 (0.5)	8 (0.1)	4 (0.1)	2 (0.3)
Diseases of the respiratory system	20 (0.4)	8 (0.5)	9 (2.5)	70 (0.7)	27 (1.0)	37 (6.0)
Chronic obstructive pulmonary disease	13 (0.3)	3 (0.2)	4 (1.1)	27 (0.3)	12 (0.4)	19 (3.1)
Influenza and pneumonia	3 (0.1)	3 (0.2)	3 (0.8)	31 (0.3)	12 (0.4)	16 (2.6)
Ischemic heart disease	81 (1.6)	28 (1.6)	19 (5.2)	182 (1.8)	69 (2.5)	83 (13.4)
Cerebrovascular disease	15 (0.3)	8 (0.5)	7 (1.9)	34 (0.3)	20 (0.7)	16 (2.6)
Diabetes	10 (0.2)	0 (0.0)	0 (0.0)	10 (0.1)	0 (0.0)	2 (0.3)
Dementia and Alzheimer's disease	0 (0.0)	0 (0.0)	1 (0.3)	2 (<0.1)	0 (0.0)	13 (2.1)
External causes, including suicide	9 (0.2)	2 (0.1)	2 (0.5)	3 (<0.1)	3 (0.1)	8 (1.3)
Suicide	6 (0.1)	0 (0.0)	0 (0.0)	0 (0.0)	1 (<0.1)	1 (0.2)
Other specified	109 (2.1)	49 (2.8)	28 (7.7)	236 (2.3)	81 (2.9)	80 (12.9)

*Number of patients alive at the beginning of the follow-up window.

^†^ Patients alive at the beginning of the follow-up window who did not die during the specified period of follow-up.

‡ Percentage of all patients alive at the beginning of the follow-up window.

### Competing-risks regression

The estimated sub-hazard ratios (SHRs) and their 95% CIs of cause-specific death within 5 years following a diagnosis of esophageal cancer from competing-risks regression are presented in Table 5. Female patients had lower risks of death from all listed causes, although the reduced risk was only statistically significant for death from esophageal cancer (SHR = 0.90, 95% CI: 0.87, 0.94). Risks of death from esophageal cancer and ischemic heart disease or cerebrovascular disease increased with age, suggesting a monotonic age-response association. Patients who were diagnosed in more recent calendar periods had lower risk of death form esophageal cancer compared with those diagnosed in earlier periods; patients diagnosed in 2001-2014 had a 30% decreased risk of death from esophageal cancer compared to those diagnosed in 1961-1971 (SHR = 0.71, 95% CI: 0.67, 0.75). We also observed a reduced risk of death from other cancers in patients diagnosed in later calendar periods, although it reached the level of statistical significance only for the period 1981-1990 compared with the earliest period 1961-1970 (SHR = 0.80, 95% CI: 0.67, 0.91).

**Table 5:** Subhazard ratios with 95% confidence intervals of cause-specific death within 5 years of follow-up by sex, age at diagnosis, and calendar period of diagnosis, from competing-risks regression

Characteristic	Causes of death				
	Esophageal cancer	Non-esophageal cancers	Ischemic heart disease or cerebrovascular disease	Respiratory diseases	Other causes
Sex					
Male	1.00 (reference)	1.00 (reference)	1.00 (reference)	1.00 (reference)	1.00 (reference)
Female	0.90 (0.87, 0.94)	0.93 (0.82, 1.04)	0.90 (0.75, 1.08)	0.82 (0.57, 1.18)	0.98 (0.83, 1.16)
Age at diagnosis, years					
< 50	1.00 (reference)	1.00 (reference)	1.00 (reference)	1.00 (reference)	1.00 (reference)
50-59	1.25 (1.13, 1.39)	0.77 (0.57, 1.05)	5.24 (1.27, 21.66)	0.67 (0.26, 1.73)	0.67 (0.41, 1.11)
60-69	1.26 (1.14, 1.39)	0.99 (0.75, 1.30)	6.74 (1.67, 27.23)	0.65 (0.27, 1.54)	0.79 (0.50, 1.25)
70-79	1.34 (1.21, 1.48)	0.96 (0.73, 1.27)	11.44 (2.85, 45.92)	1.15 (0.50, 2.65)	1.24 (0.79, 1.93)
≥ 80	1.66 (1.50, 1.85)	0.83 (0.62, 1.10)	16.26 (4.05, 65.37)	1.33 (0.56, 3.14)	1.83 (1.17, 2.87)
Calendar period of diagnosis					
1961-1970	1.00 (reference)	1.00 (reference)	1.00 (reference)	1.00 (reference)	1.00 (reference)
1971-1980	1.08 (1.02, 1.16)	0.94 (0.78, 1.13)	0.60 (0.43, 0.84)	0.54 (0.25, 1.15)	0.57 (0.41, 0.79)
1981-1990	0.96 (0.91, 1.03)	0.80 (0.67, 0.97)	1.20 (0.91, 1.58)	1.86 (1.06, 3.28)	0.72 (0.54, 0.97)
1991-2000	0.80 (0.75, 0.85)	0.91 (0.76, 1.08)	0.97 (0.73, 1.28)	1.30 (0.73, 2.32)	1.14 (0.88, 1.47)
2001-2014	0.71 (0.67, 0.75)	0.91 (0.78, 1.07)	0.69 (0.53, 0.90)	1.28 (0.73, 2.22)	1.10 (0.87, 1.40)

**Figure 1:**
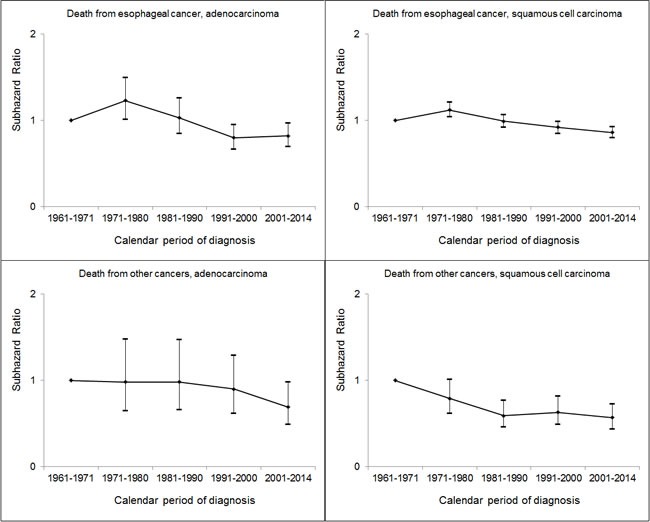
Risks of death from esophageal cancer and other cancers in patients diagnosed with adenocarcinoma and squamous cell carcinoma of the esophagus as estimated as sub-hazard ratios (95% confidence intervals) from competing-risks regression

We performed stratified analyses by histological type for deaths from esophageal cancer and non-esophageal cancers, but not by other variables due to the limited number of deaths from other causes. The results are shown in Table 6. The reduced risk of death from esophageal cancer in female patients was limited to those diagnosed with ESCC (SHR = 0.85, 95% CI: 0.81, 0.89) and not those with EAC (SHR = 0.97, 95% CI: 0.88, 1.06). There was an increased risk of death from esophageal cancer with older age in patients diagnosed with both ESCC and EAC. Regardless of histological type, patients diagnosed with esophageal cancer in later calendar periods had lower risks of death from both esophageal cancer and other cancers, compared with those diagnosed in earlier periods (Figure 1).

**Table 6:** Subhazard ratios with 95% confidence interval of death from esophageal cancer and non-esophageal cancers within 5 years of follow-up by histological type, from competing-risks regression

Characteristic	Adenocarcinoma		Squamous cell carcinoma	
	Esophageal cancer	Non-esophageal cancers	Esophageal cancer	Non-esophageal cancers
Sex				
Male	1.00 (reference)	1.00 (reference)	1.00 (reference)	1.00 (reference)
Female	0.97 (0.88, 1.06)	1.19 (0.98, 1.45)	0.85 (0.81, 0.89)	0.93 (0.78, 1.12)
Age at diagnosis, years				
< 50	1.00 (reference)	1.00 (reference)	1.00 (reference)	1.00 (reference)
50-59	1.19 (0.99, 1.43)	0.68 (0.45, 1.04)	1.13 (0.99, 1.29)	1.16 (0.65, 2.04)
60-69	1.09 (0.91, 1.30)	0.96 (0.66, 1.40)	1.18 (1.03, 1.34)	1.43 (0.84, 2.45)
70-79	1.26 (1.06, 1.50)	0.85 (0.57, 1.23)	1.23 (1.08, 1.40)	1.46 (0.85, 2.49)
≥ 80	1.66 (1.38, 1.99)	0.72 (0.48, 1.06)	1.49 (1.30, 1.70)	1.11 (0.63, 1.95)
Calendar period of diagnosis				
1961-1970	1.00 (reference)	1.00 (reference)	1.00 (reference)	1.00 (reference)
1971-1980	1.23 (1.01, 1.50)	0.98 (0.65, 1.48)	1.12 (1.04, 1.21)	0.79 (0.62, 1.01)
1981-1990	1.03 (0.85, 1.26)	0.98 (0.66, 1.47)	0.99 (0.92, 1.07)	0.59 (0.46, 0.77)
1991-2000	0.80 (0.67, 0.95)	0.90 (0.62, 1.29)	0.92 (0.85, 0.99)	0.63 (0.49, 0.82)
2001-2014	0.82 (0.70, 0.97)	0.69 (0.49, 0.98)	0.86 (0.80, 0.93)	0.57 (0.44, 0.73)

### Cox regression

The hazard ratios from the Cox regression were similar to the SHRs from competing-risks regression, except for that the observed associations from Cox regression were generally stronger than those from competing-risks regression (Supplementary Tables 1 and 2).

## DISCUSSION

This study revealed that although patients diagnosed with esophageal cancer were more likely to die from esophageal cancer than other causes, there were also other major causes of death, including non-esophageal cancers, ischemic heart disease, cerebrovascular disease and respiratory diseases. Female patients had a lower risk of death from esophageal cancer than male patients, which was more pronounced in patients with adenocarcinoma. Risks of death from esophageal cancer and other cancers were lower in patients diagnosed in more recent calendar periods compared to those diagnosed in earlier periods.

To the best of our knowledge, this study is the first population-based study and has the largest sample size on causes of death in patients with esophageal cancer. The complete nationwide coverage and high accuracy of the Swedish Cancer Register and the Swedish Cause of Death Register ensured the representativeness of the findings and minimized selection bias. In addition, the competing-risks regression employed in this study posits a model for the sub-hazard function of a failure event in the presence of competing failure events, i.e. risk of death from a specific cause of death of interest in the presence of other competing causes in the context of mortality data. On the contrary, the alternative Cox regression treats competing causes of death as censored events, but such events are in fact distinct from standard censorings due to loss to follow-up, merely obstructing researchers from observing the events of interest. [11, 12] Although both methods yielded similar estimates in this study, findings from ordinary Cox regression need to be interpreted with more caution, given the possibly biased estimates of cause-specific risk of death by failing to account for competing causes. [11–13] As they were performed in previous causes-of-death analyses, [14–16] we analyzed the primary causes of death only from death records and did not consider other contributing causes due to methodological constraints, although the assumption that each death is caused by only one disease is debatable. [17] In addition, despite the relatively large sample size compared with previous studies, this study did not have adequate statistical power for all subgroup analyses on non-cancer causes of death by histological type. Analysis by stage was not within the scope of this study.

The literature examining causes of death in patients diagnosed with esophageal cancer is limited. Current knowledge is based on hospital-based studies with hundreds or fewer patients in the selected group who underwent esophagectomy. [6–8, 18] These studies showed that esophageal cancer was the leading cause of death in patients diagnosed with this cancer, and other major causes of death were non-esophageal cancers, cardiovascular disease and respiratory diseases. It has also been suggested that gastric, head and neck, and lung cancers were the most common causes of death among deaths caused by non-esophageal malignancies. These observations are consistent with the findings of the present study. However, no previous studies have assessed the temporal trends in the cause-specific risks of death or had the statistical power to conduct robust subgroup analyses.

This study suggests that esophageal cancer remains the predominant cause of death in patients diagnosed with this cancer, particularly within the first year of diagnosis. A large study from the Surveillance, Epidemiology, and End Results Program (SEER) database in the United States has shown that the esophageal cancer-specific survival was around only 10% in patients with metastatic esophageal cancer 2 years after diagnosis, whereas it was over 20% in patients with localized esophageal cancer 5 years after diagnosis. [19] Therefore, death directly from esophageal cancer may be more relevant to patients who are diagnosed at advanced stages, while patients who have curative therapies also face considerable risk of death from this cancer due to postoperative complications or tumor recurrence. The observed decreased risk of death from esophageal cancer in patients diagnosed with esophageal cancer in recent calendar periods might be attributable to a better selection of patients suitable for curative surgery, increased use of neoadjuvant therapies, as well as increased centralization of the treatment. [4, 20] Despite all efforts to improve the diagnostic procedures and therapy, the overall 5-year survival following diagnosis in patients with esophageal cancer remains lower than 20% even in Western societies. Tumor stage at diagnosis remains by far the strongest prognostic factor. [3–5] Thus, earlier tumor detection at a more curable stage would likely improve the prognosis in patients with esophageal cancer. Upper endoscopy provides an opportunity of early detection of esophageal cancer or its precursors, i.e., squamous-cell dysplasia for ESCC and Barrett's esophagus with dysplasia for EAC, although a universal strategy in the general population is not feasible due to the low incidence of esophageal cancer. [21, 22] Identifying high-risk individuals based on their risk factor profiles is a promising approach, which requires reliable tools of risk stratification in the population. A few risk prediction models combining information on readily identifiable risk factors have been developed for the selection of individuals with high risk of esophageal cancer, but remain to be further validated. [23, 24]

The present study shows considerable risk of death from non-esophageal cancers, particularly from cancers of the digestive organs, head and neck, and lung. These results are in line with analyses from the SEER database in the United States which revealed increased risks of secondary malignancies, including cancers of oral and pharynx, stomach, and lung, following a diagnosis of esophageal cancer. [9, 10] These findings may be explained by shared risk factors for esophageal and non-esophageal malignancies, e.g. tobacco smoking, alcohol abuse and inappropriate diet, but are also possibly related to treatments administered to patients with esophageal cancer, e.g. irradiative effects from radiotherapy. The observed lower risk of death from esophageal cancer in female patients than in males was more pronounced in patients with ESCC and is consistent with some previous evidence. [19] However, the underlying mechanisms for a protective role of the female sex are unclear. It has been hypothesized that sex hormones may play a role in both the development and prognosis of esophageal cancer, but existing evidence remains inconclusive and warrants further research. [25]

Ischemic heart disease, cerebrovascular disease and respiratory diseases are increasingly common causes of death in the general population, especially in an era where infectious diseases are replaced by chronic diseases. [26, 27] Patients with esophageal cancer may be more vulnerable to death from these conditions due to increased rates of immunodeficiency, malnutrition and systematic inflammation. [28, 29] Modifications of lifestyle risk factors, e.g. smoking cessation, physical activity, and dietary habits, have been suggested to be associated with reduced all-cause and certain cause-specific mortality. [30–32] However, benefits of such lifestyle changes on the prognosis in patients with esophageal cancer are less clear. [29] More studies, preferably well-designed randomized controlled trials with sufficient statistical power, are needed to explore and assess effective interventions aiming at reducing overall and cause-specific mortality in patients with esophageal cancer.

In summary, this nationwide and population-based study reveals that patients diagnosed with esophageal cancer are more likely to die from this cancer than from any other causes. However, these patients also face considerable risk of death from other causes, particularly from non-esophageal cancers, ischemic heart disease, cerebrovascular disease and respiratory diseases. More efforts in identifying high-risk individuals for early detection, refining the selection of patients for tailored treatment, exploring new treatment strategies, and assessing effective lifestyle interventions, are needed to improve the overall and cause-specific survival in patients diagnosed with esophageal cancer.

## MATERIALS AND METHODS

### Study design and population

This study was a population-based cohort study. The study population consisted of all patients who were diagnosed with esophageal cancer (International Classification of Diseases, 7^th^ edition [ICD-7] code: 150) in Sweden between 1^st^ January 1961 and 31^st^ December 2014. These patients were identified from the Swedish Cancer Registry, which has a 98% nationwide coverage of esophageal cancer and 96% coverage of all cancers. [33, 34] We obtained information on causes of death of the cohort members through linkage to the Swedish Cause of Death Register with the unique personal identity number. The Cause of Death Register covers all deaths of persons who were registered in Sweden in the year they died, and the non-reporting rate was estimated as lower than 1%. [35] All participants were followed up from the date of esophageal cancer diagnosis until the date of death or the end of the study (31^st^ December 2014), whichever occurred first. This study was approved by the Regional Ethical Review Board in Stockholm, Sweden (Protocol Number: 2010/727-31/2).

### Outcome ascertainment

We described the distribution of major cause of death, which accounts for over 95% of all deaths in the study population. The specific ICD codes for different causes of death are listed in Supplementary Table 3. All causes of death were further categorized into the following five groups: (1) esophageal cancer, (2) other (non-esophageal) cancers, (3) ischemic heart disease or cerebrovascular disease, (3) diseases of the respiratory system and (5) other causes. Only the primary causes of death, i.e. the disease or injury initiating the train of events leading directly to death, or the circumstances of the accident or violence producing the fatal injury, were analyzed in this study.

### Statistical analysis

We assessed the distribution of primary major causes of death in patients with esophageal cancer by categories of duration of follow-up ( ≤ 1, 1-5, or > 5 years), which was calculated as the date of diagnosis to the end of follow-up. We used competing-risks regression embedded in the statistical software STATA (version 14; http://www.stata.com/features/overview/competing-risks-regression/) to compare the cause-specific risks of death in patients with different characteristics. The competing-risks regression is based on the method of Fine and Gray and estimates the cause-specific SHRs in the presence of competing risks, i.e., deaths from other causes. [11, 36] Variables included in the model were sex (male or female), age at diagnosis ( < 50, 50-59, 60-69, 70-79 or ≥ 80 years), and calendar period of diagnosis (1961-1970, 1971-1980, 1981-1990, 1991-2000 or 2001-2014). For comparison purposes, we also performed Cox proportional hazard regression where competing events were considered as censored observations. We further performed stratified analyses by histological type. All statistical analyses except for the competing-risks regression were performed using IBM SPSS Statistics for Windows, version 23 (Armonk, NY: IBM Corp.).

## SUPPLEMENTARY MATERIALS TABLES


