# Supplementary material for: Cause of death in patients diagnosed with esophageal cancer in Sweden: a population-based study
Source: Oncotarget. 2017 Feb 11;8(31):51800–9. doi: 10.18632/oncotarget.15270 (PMC5584290; doi:10.18632/oncotarget.15270)
Supplement: Supplementary file 1 [file oncotarget-08-51800-s001.docx]

| **Supplementary Table 1.** Hazard ratios with 95% confidence interval of cause-specific death by sex, age at diagnosis, and calendar period of diagnosis, from Cox regression | | | | | |
| --- | --- | --- | --- | --- | --- |
| Characteristic | Causes of death | | | | |
|  | Esophageal cancer | Non-esophageal cancers | IHD/CVD | Respiratory diseases | Other causes |
| Sex |  |  |  |  |  |
| Male | 1.00 (reference) | 1.00 (reference) | 1.00 (reference) | 1.00 (reference) | 1.00 (reference) |
| Female | 0.85 (0.82, 0.89) | 0.79 (0.71, 0.88) | 0.76 (0.64, 0.89) | 0.62 (0.46, 0.84) | 0.88 (0.76, 1.02) |
| Age at diagnosis, years |  |  |  |  |  |
| < 50 | 1.00 (reference) | 1.00 (reference) | 1.00 (reference) | 1.00 (reference) | 1.00 (reference) |
| 50-59 | 1.28 (1.15, 1.42) | 0.90 (0.68, 1.17) | 4.82 (1.94, 11.99) | 1.10 (0.53, 2.26) | 0.86 (0.57, 1.28) |
| 60-69 | 1.35 (1.22, 1.49) | 1.22 (0.96, 1.57) | 7.47 (3.06, 18.20) | 1.25 (0.63, 2.46) | 1.18 (0.82, 1.70) |
| 70-79 | 1.55 (1.40, 1.71) | 1.42 (1.11, 1.82) | 14.39 (5.93, 34.94) | 2.35 (1.20, 4.58) | 2.28 (1.59, 3.26) |
| ≥ 80 | 2.19 (1.98, 2.43) | 1.61 (1.24, 2.10) | 28.15 (11.52, 68.81) | 4.12 (2.03, 8.33) | 4.38 (3.03, 6.35) |
| Calendar period of diagnosis |  |  |  |  |  |
| 1961-1970 | 1.00 (reference) | 1.00 (reference) | 1.00 (reference) | 1.00 (reference) | 1.00 (reference) |
| 1971-1980 | 1.01 (0.95, 1.08) | 0.99 (0.83, 1.18) | 0.74 (0.56, 0.98) | 1.08 (0.61, 1.91) | 0.73 (0.56, 0.97) |
| 1981-1990 | 0.91 (0.86, 0.97) | 0.84 (0.70, 1.00) | 1.17 (0.91, 1.49) | 1.89 (1.14, 3.14) | 0.93 (0.72, 1.20) |
| 1991-2000 | 0.72 (0.68, 0.76) | 0.77 (0.65, 0.91) | 0.77 (0.60, 0.99) | 1.31 (0.79, 2.18) | 1.08 (0.86, 1.36) |
| 2001-2014 | 0.63 (0.59, 0.66) | 0.72 (0.62, 0.85) | 0.52 (0.40, 0.66) | 1.19 (0.72, 1.96) | 0.97 (0.77, 1.21) |
| CVD: cerebrovascular disease; IHD: ischemic heart disease. | | | | | |

| **Supplementary Table 2.** Hazard ratios with 95% confidence interval of death from esophageal cancer and non-esophageal cancers within 5 years of follow-up by histological type, from Cox regression | | | | | |
| --- | --- | --- | --- | --- | --- |
| Characteristic | Adenocarcinoma | |  | Squamous cell carcinoma | |
|  | Esophageal cancer | Non-esophageal cancers |  | Esophageal cancer | Non-esophageal cancers |
| Sex | | | | | |
| Male | 1.00 (reference) | 1.00 (reference) |  | 1.00 (reference) | 1.00 (reference) |
| Female | 0.98 (0.90, 1.07) | 1.12 (0.93, 1.35) |  | 0.78 (0.74, 0.82) | 0.73 (0.62, 0.86) |
| Age at diagnosis, years | | | | | |
| < 50 | 1.00 (reference) | 1.00 (reference) |  | 1.00 (reference) | 1.00 (reference) |
| 50-59 | 1.15 (0.95, 1.39) | 0.74 (0.50, 1.10) |  | 1.20 (1.05, 1.38) | 1.04 (0.67, 1.61) |
| 60-69 | 1.11 (0.93, 1.33) | 1.07 (0.75, 1.53) |  | 1.31 (1.15, 1.49) | 1.36 (0.90, 2.04) |
| 70-79 | 1.38 (1.15, 1.65) | 1.16 (0.81, 1.66) |  | 1.49 (1.31, 1.70) | 1.66 (1.10, 2.50) |
| ≥ 80 | 2.10 (1.75, 2.53) | 1.38 (0.94, 2.02) |  | 2.05 (1.79, 2.35) | 1.59 (1.02, 2.49) |
| Calendar period of diagnosis | | | | | |
| 1961-1970 | 1.00 (reference) | 1.00 (reference) |  | 1.00 (reference) | 1.00 (reference) |
| 1971-1980 | 1.11 (0.92, 1.34) | 1.08 (0.73, 1.61) |  | 1.03 (0.96, 1.11) | 0.86 (0.69, 1.09) |
| 1981-1990 | 0.96 (0.80, 1.15) | 1.04 (0.71, 1.52) |  | 0.92 (0.86, 0.99) | 0.66 (0.52, 0.83) |
| 1991-2000 | 0.68 (0.57, 0.80) | 0.73 (0.51, 1.03) |  | 0.81 (0.76, 0.88) | 0.59 (0.46, 0.74) |
| 2001-2014 | 0.68 (0.58, 0.79) | 0.57 (0.41, 0.80) |  | 0.77 (0.72, 0.83) | 0.54 (0.43, 0.69) |

| **Supplementary Table 3.** ICD codes for causes of death | | | | |
| --- | --- | --- | --- | --- |
| Causes | ICD-7 | ICD-8 | ICD-9 | ICD-10 |
| Esophageal cancer | 150 | 150 | 150 | C15 |
| Cancers other than the esophageal cancer | 140-209, excluding 150 | 140-209, excluding 150 | 140-208, excluding 150 | C00-C99, excluding C15 |
| Infectious and parasitic diseases | 001-138 | 001-136 | 001-139 | A00-B99 |
| Diseases of the respiratory system | 470-527, 241 | 460-519 | 460-519 | J00-J99 |
| Chronic obstructive pulmonary disease | 501, 502, 526, 527, 241 | 490-493, 518 | 490-494, 496 | J40-J44 |
| Influenza and Pneumonia | 480-483, 490-493 | 470-474, 480-483, 485-486 | 480-487 | J09-J18 |
| Ischemic heart disease | 420 | 410-414 | 410-414 | I20-I25 |
| Cerebrovascular disease | 330-334 | 430-434, 436-438 | 430-434, 436-438 | I60-I69 |
| Diabetes | 260 | 250 | 250 | E10-E14 |
| Dementia and Alzheimer’s disease | 304-306 | 290, 293 | 290, 331 | F00, F01,F02, F03, G30 |
| External causes, including suicide | E800-E999 | E800-E999 | E800-E999 | V01-Y98 |
| Suicide | E971-E979 | E950-E959 | E950-E959 | X60-X84 |
| Other specified ^*^ | 001-795 | 000-796 | 001-799 | A00-R99 |
| ICD: International Classification of Diseases  ^*^ Codes of all other listed causes should be excluded. | | | | |
